# Antifreeze proteins produced by Antarctic yeast from the genus *Glaciozyma* as cryoprotectants in food storage

**DOI:** 10.1371/journal.pone.0318459

**Published:** 2025-03-06

**Authors:** Edyta Majewska, Aleksandra Twarda-Clapa, Marzena Jędrzejczak-Krzepkowska, Anna Kamińska-Dwórznicka, Małgorzata Zakłos-Szyda, Aneta Monika Białkowska

**Affiliations:** 1 Faculty of Biotechnology and Food Sciences, Institute of Molecular and Industrial Biotechnology, Lodz University of Technology, Lodz, Poland; 2 Faculty of Food Sciences, Department of Food Engineering and Process Management, Warsaw University of Life Sciences, Warsaw, Poland; The University of Tennessee Health Science Center, UNITED STATES OF AMERICA

## Abstract

Synthesis of antifreeze proteins (AFPs) is one of the adaptations of psychrophilic yeast to live in cold environments. AFPs demonstrate thermal hysteresis (TH) activity and inhibit the recrystallization of ice (IRI) during periodic temperature fluctuations. In this study, the Antarctic yeast strain 186, identified as *Glaciozyma martinii*, was found to synthesize an extracellular, glycosylated ~27 kDa ice-binding protein (GmAFP) exhibiting IRI activity. It is the first evidence of AFP secretion by the psychrophilic yeast *Glaciozyma martinii*. To scale up protein production, a synthetic gene from a closely related cold-adapted species, *Glaciozyma antarctica*, was expressed in *Pichia pastoris* GS115 strain. The recombinant 26.57 kD protein (GaAFP) displayed IRI activity and a cryoprotective effect in food storage. The addition of GaAFP to the stored frozen vegetables and fruits (carrot, kohlrabi, and blueberry) markedly reduced the drip loss during the thawing process and positively affected their structure, with an effect similar to glycerol. Moreover, GaAFP increased the cell survival of *Saccharomyces cerevisiae* after freezing. The insights from this study provided proof that AFPs from natural sources may serve as competent biodegradable, eco-friendly, non-cytotoxic and biocompatible substitutes for traditional cryoprotectants in enhancing the quality of frozen foods.

## Introduction

Psychrophiles, adapting to life at low temperatures, have developed numerous defense mechanisms that are intriguing subjects for research in the fields of molecular biology and biotechnology [1]. The optimal temperature for the growth of psychrophiles is 15°C, but some can thrive and reproduce even at − 15°C, with their metabolism remaining active down to −25°C [2]. Apart from producing psychrozymes and cold shock proteins (CSPs), psychrophilic organisms can synthesize cryoprotectants such as trehalose or antifreeze proteins (AFPs) [3].

This study focuses on AFPs, which are characterized by two key activities: thermal hysteresis (TH) and ice recrystallization inhibition (IRI) [4]. TH refers to the difference between water’s melting and freezing points. AFPs adsorb onto the surface of nucleated ice crystals, preventing their growth and lower the freezing point of the solution [5]. Ice recrystallization is the process whereby larger ice crystals form at the expense of smaller ones, potentially damaging the cells. AFPs inhibit this process even at extremely low concentrations (0.27 µ M of AFP isoform Tis8 from snow-mould *Typhula ishikariensis*), protecting against freezing during temperature fluctuations [6]. AFP distribution spans various psychrophilic organisms, with well-characterized AFPs isolated from fish, plants, insects, and bacteria. Notably, only two species of yeast (*Glaciozyma antarctica* and *Glaciozyma* sp. AY30) and three species of mould fungi (*Typhula ishikariensis*, *Antarctomyces psychrotrophicus*, and *Coprinus psychromorbidus*) have been reported to produce these proteins [7–10]. The diversity in AFP structures arises from their independent evolutionary origins and the heterogeneity of the surface of their ligand— the ice [11].

Due to their properties, AFPs are utilized in multiple industries, including agriculture (for temperature-resistant crop plants) [12], cryopreservation (for long-term storage of tissues and cells) [13,14], and material technology (deicing refrigerated surfaces) [15,16]. The food industry, in particular, extensively employs AFP proteins to improve food quality during freezing [17]. Freezing is one of the most common methods used to prolong the shelf life of many food items. It also enables the utilization of seasonal fruits and vegetables during periods when they are typically unavailable. AFPs were also tested for improving the structure of ice cream, frozen dough, or frozen beef [18–20]. Consequently, the challenge lies in executing this process correctly to prevent the formation and growth of ice crystals (ice recrystallization) or their multiplication (ice nucleation) [21]. Cryoprotective agents (CPAs) like glycerol, sucrose, trehalose, or salt are added to enhance the quality of stored food. However, the drawback of these additives is the modification of the taste. Hence, the quest for alternative cryoprotectant sources has commenced, which includes exploring AFPs.

The discovery of AFPs and their unique activities has generated an ongoing interest, with the market for these proteins projected to reach $50 million by 2028, up from $10 million in 2023 (according to expert forecasts from MarketsAndMarkets™ INC) [22]. The primary drivers of this growth are the food and medical industries, necessitating continuous optimization of large-scale production of native or recombinant AFPs [15,17,23,24].

The collection of Antarctic microorganisms at the Institute of Molecular and Industrial Biotechnology of Lodz University of Technology gathers a variety of psychrophilic yeast, bacterial and fungal species. One of them is the yeast strain *Glaciozyma martinii* 186, which secretes a glycosylated AFP protein with IRI activity (referred to as GmAFP). Unfortunately, the native strain produces this protein in low concentrations, and its synthesis requires a minimum of 14 days under refrigeration. Consequently, the homologous *afp* gene from *Glaciozyma antarctica* was cloned into pPICZB vector and expressed in *Pichia pastoris* GS115 strain. The IRI activity of this recombinant protein (referred to as GaAFP) was confirmed through microscopic analysis of IRI process. Furthermore, we provide the first evidence that the addition of the recombinant GaAFP has a cryoprotective effect comparable or even better than the popular cryoprotectant—glycerol, which was demonstrated during the storage of frozen vegetables and fruits or baker’s yeast cells (*Saccharomyces cerevisiae*).

## Materials and methods

### Psychrophilic yeast identification

The psychrophilic yeast, identified as *Glaciozyma martinii* 186, was originally isolated from Henryk Arctowski Polish Antarctic Station on King George Island (62°09′41″ S, 58°28′10″ W) and is a part of the collection of Antarctic microorganisms at the Institute of Molecular and Industrial Biotechnology, Lodz University of Technology. Its taxonomic classification was confirmed by sequencing the D1/D2 regions of the 26S rDNA domain and the ITS1–5.8S–ITS2 regions. Sequences were deposited in GenBank with the accession numbers KX640899 and KX640897. Additionally, the analysis of phylogenetic marker sequences of other psychrophilic yeasts of the same genus available in the GenBank database was conducted. To construct the phylogenetic tree, a multiple sequence alignment (MSA) was performed using the ClustalW algorithm in MEGA11 Molecular Evolutionary Genetics Analysis version 1136 [25]. After the alignment, sequences were manually edited. The Maximum Parsimony statistical method and a bootstrap phylogeny test with a replication count of 500 were chosen to conduct the phylogenetic analysis.

### Native GmAFP from *Glaciozyma martinii* 186—isolation and purification

The strain was cultured on Nutrient Broth (NB) plates (containing bactopeptone 15.0 g/L, yeast extract 3.0 g/L, glucose 1.0 g/L, sodium chloride 6.0 g/L, pH 7.5) for 14 days at 6°C. Subsequently, a large-scale (2 L) culture was conducted for 14 days at 4°C with shaking at 180 rpm. After cultivation, post-culture liquid was separated from the yeast cells by centrifugation (10000 rpm, 30 min, 4°C). The purification process involved two stages. Firstly, the post-culture liquid was concentrated using tangential flow filtration (TFF) with a Sartorius Stedim system and a Hydrostat membrane with a cutoff of 10 kDa (Sartorius, Göttingen, Germany). The second step of protein purification included ice affinity chromatography using laboratory-assembled equipment (S1 Fig) [26]. In addition, the status of glycosylation was determined for the purified GmAFP protein using the commercial Pierce Glycoprotein Staining Kit (ThermoFisher Scientific, Waltham, MA, USA) based on the Periodic Acid-Schiff (PAS) method [27].

### Vector construction, protein expression and purification of recombinant GaAFP from *Glaciozyma antarctica*

The *afp* gene sequence coding for Afp4 protein (referred to as GaAFP) from *Glaciozyma antarctica* PI12 (GenBank: JF412502.1) was synthesized by GeneArt (Thermo Fisher Scientific, Regensburg, Germany). The codon sequence was optimized for gene expression in a heterologous mesophilic yeast system (S1 Table). For expression, it was decided to utilize an expression vector pPICZB designed for the *Pichia pastoris* strains. The *afp* gene sequence was amplified using high-fidelity polymerase (Q5® High-Fidelity 2X Master Mix, New England Biolabs, Ipswich, MA, USA) with primers FWDEcoRIIpPICZ and REVKpnIpPICZ (S2 Table). The accuracy of cloning topPICZB was verified by sequencing at an external facility (Genomed, Warsaw, Poland) using 5′AOX1 and 3′AOX1 primers (S2 Table ).

The expression vector pPICZB carrying the *afp* gene was linearized for transformation into *Pichia pastoris* GS115 host strain via electroporation. PmeI restriction enzyme (ThermoFisher Scientific, Waltham, MA, USA) was used to digest the pPICZB vector according to manufacturer’s instructions. Electroporation was performed using a MicroPulser system (Bio-Rad, Hercules, CA, USA) with a dedicated Pic program, employing settings of 1 pulse at 2.0 kV for 5 msec with a 0.2 cm cuvette. After the pulse, cells were regenerated in 1 M cold sorbitol at 28°C for 1 h. Transformants were selected on YPDS plates (containing 10 g/L yeast extract, 20 g/L peptone, 20 g/L dextrose, 182.2 g/L sorbitol, and 20 g/L agar) supplemented with 100 µg/mL of zeocin. Positive transformants were confirmed via colony PCR using FWDEcoRIpPICZ and REVKpnIpPICZ primers, as well as 5′AOX1 and 3′AOX1 primers. In parallel, transformation and expression controls were conducted using transformants carrying the pPICZB plasmid without the *afp* gene.

The positive transformant was precultured overnight on YPD medium (containing 10 g/L yeast extract, 20 g/L peptone, and 20 g/L dextrose) supplemented with 100 µg/mL of zeocin at 28°C with shaking (200 rpm). Subsequently, 25 mL of BMGY medium (containing 1% yeast extract, 2% peptone, 0.1 M potassium phosphate buffer pH 6.0, 1.34% Yeast Nitrogen Base (YNB), and 1% glycerol) was inoculated with 50 µl of preculture and cultivated for 48 h at both 20°C and 28°C with shaking (200 rpm). After two days, the culture was centrifuged under sterile conditions and transferred to BMMY medium (containing 1% yeast extract, 2% peptone, 0.1M potassium phosphate buffer pH 6.0, 1.34% YNB) containing either 1% or 0.5% methanol. The expression was carried out for 72 h at either 20°C or 28°C, with shaking (200 rpm), and addition of 1% or 0.5% methanol every 12 h. The supernatant was collected after 48 and 72 h by centrifugation (5000g, 4°C, 5 min), and the expressed proteins were visualized by SDS-PAGE using a 12% acrylamide gel. The optimal expression condition was determined by Bradford method.To purify the recombinant GaAFP protein for further experiments, the scale of the *Pichia pastoris* culture was increased to 2L (growth in 1 L unbaffled flasks filled to 25% volume for adequate aeration) and cultivated in experimentally determined optimal conditions: (28°C, 72 h, 1% methanol induction.

The purification of the recombinant GaAFP involved two steps. The supernatant (post-culture liquid) was collected by centrifugation (10000 rpm, 30 min, 4°C) and subjected to the same purification procedure as for the native protein GmAFP (concentration by TFF and ice affinity chromatography).

### Microscopic analysis of the ice recrystallization inhibition (IRI)

The IRI activity was assessed using an optical microscope (Nikon Alpha Phot-2) equipped with a camera for photo recording (Nikon DS-Fi1) and a cooling stage (Linkam Scientific PE 94), allowing the sample to remain frozen [28,29]. The inhibition activity was evaluated for both native GmAFP and recombinant GaAFP. A negative control sample consisted of a 50% sucrose solution. In brief, 40 µ L of appropriately diluted sample (protein solution diluted to 0.001% and mixed 1:1 with 50% sucrose) was sandwiched between two coverslips on a glass slide, covered with another coverslip, and sealed with silicone. This sample was flash-frozen in liquid nitrogen for rapid transition from liquid to glassy state. Subsequently, the samples were transferred to a microscope cooled to −8°C, and images were captured at 0, 10, 20, 30, 40, 50, and 60 min. The acquired images were analyzed using NIS Elements D software (Nikon, Japan). Approximately 300 crystals were marked from each sample, and parameters such as area, equivalent diameter, and standard deviation were calculated using NIS Elements D Imaging software (ver. 5.00, Nikon, Japan) [30]. Additionally, the frequency distribution of ice crystal size was determined using MS Excel’s data macro-analysis tool (Microsoft Corporation, Redmond, WA, USA). The relative frequency of each class interval was calculated as the number of crystals in the class (class frequency) divided by the total number of crystals and expressed as percentages. The minimum and maximum ice crystal sizes, mean diameter (DA), and standard deviation for each class were also computed (S3 Table). The size distribution of the ice crystals was illustrated using graphs generated in MS Excel (Microsoft Corporation, Redmond, WA, USA). Based on this analysis, the parameter X_50_ was determined, representing the ice crystal diameter at 50% of the cumulative distribution of the sample.

### Cell structure of vegetables and fruit

To evaluate the cryoprotective impact of GaAFP, microscopic visualization of cellular structures after freezing was conducted. Two vegetables, carrots (*Daucus carota* L., variety Fantazja) and kohlrabi (*Brassica oleracea* var. *gongylodes* L., variety Di Vienna bianco), and one fruit, blueberry (*Vaccinium corymbosum,* variety Bluecrop), were selected for structural analysis. The plant material was purchased from the provider on the local market who provided the information about the varieties and the place and time of the harvest (August 2023 in central Poland). No genetically modified plants were used in this study. The experiment complied with institutional, national, and international guidelines and legislation. The vegetables and fruits were washed, peeled (in the case of carrots and kohlrabi), and dried. Small pieces of vegetables were cut, and whole blueberries were frozen with the addition of 0.1 mg/mL of expressed AFP for 16 h at‒20°C. For comparison, positive control samples (0.5M glycerol) and negative control samples (freezing without cryoprotectant) were also prepared. After overnight freezing, the samples were thawed at 4°C in a refrigerator. The small pieces of carrot and kohlrabi were washed with fresh distilled water and dried, while the blueberries were cut (with the peel observed under the microscope), and the samples were transferred to a microscope slide with a drop of water and covered with a coverslip. Observations were made immediately after preparing the slides, using a × 200 magnification for visualization with a microscope (Olympus BX51, Japan) equipped with a camera (Olympus SC50, Japan). The microscopic analyses were performed in triplicates, out of which one image per condition was presented in the article.

### Drip loss analysis

The same plant material (two vegetables, carrots and kohlrabi, and one fruit, blueberry) sourced from the local market as described in the paragraph above about the structural analysis experiment was utilized for drip loss analysis. No genetically modified plants were used in this study. The experiment complied with institutional, national, and international guidelines and legislation. In this study, the vegetables were washed, peeled, and cut into pieces measuring approximately 0.5 × 0.5 × 0.5 cm. On the contrary, blueberries were solely washed and tested whole, with fruits of similar size and weight selected for the experiment. Prior to treatment with 0.1 mg/mL GaAFP (test sample) or 0.5 M glycerol (positive control sample), all samples were weighed. Negative control samples remained untreated. Subsequently, samples were incubated at 4°C for various time intervals: 4 h, 1 day, 2 days, 4 days, and 7 days, to assess the effect of soaking on the percentage of drip loss. Following the designated soaking time, the samples were frozen at ‒20°C for 40 days. After thawing, samples were dried and weighed at room temperature. Drip loss analyses were conducted in five replicates.

Percent of drip loss for vegetables and fruit was measured using formula below


$$\%ofdriploss=\frac{initialmass\left[g\right]-finalmass\left[g\right]}{initialmass\left[g\right]}\times 100\%$$

### Viability test of frozen *Saccharomyces cerevisiae* yeast

Lyophilised baker’s yeast (*Saccharomyces cerevisiae*, 1 g) were activated for 6 hat 28°C in 10mL of YPG liquid media with shaking at 200 rpm. Firstly, the baseline concentration of viable cells was calculated using Thoma cell counting chamber with an optical microscope (Olympus BX51, Japan). The microscope preparation was stained using methylene blue dye at a concentration of 0.01% [31]. The negative control consisted of yeast frozen without any cryoprotectant, the positive control included the additive of 0.5 M glycerol, whereas the test sample—recombinant GaAFP protein (0.1 mg/mL and 0.2 mg/mL). The samples were frozen at −20°C for 35 days. After thawing, the number of viable cells was determined and the samples were examined under the microscope. The analyses were conducted in triplicates.

The % of cell survival rate was determined by the following formula


$$\%viablecells=\frac{numberofviablecellsafterfreezingproces\left[cells/mL\right]}{initialnumberofviablecells\left[cells/mL\right]}\times 100\%$$

### Cell cultures

All cell culture reagents were obtained from Life Technologies (Carlsbad, CA, USA). All experiments with cell lines were performed in a humidified 5% CO_2_ and 95% atmosphere at 37°C. All measurements were performed using the Synergy 2 BioTek Microplate Reader (BioTek, Winooski, VT, USA).

Human colon adenocarcinoma Caco-2 and HT-29, and mouse macrophage-like RAW 264.7 cell lines were obtained from ATCC (Manassas, VA, USA). Caco-2 and HT-29 cells were grown in DMEM with a 10% fetal bovine serum (FBS) medium supplemented with 100 U/mL penicillin, 100 µg/mL streptomycin, and 25 µg/mL amphotericin B. RAW 264.7 cells were maintained in DMEM medium supplemented with 10% bovine calf serum (BCS) and 100 U/mL penicillin, 100 µg/mL streptomycin, and 25 µg/mL amphotericin.

### Effect of GaAFP on the metabolic activity of cells and nitic oxide secretion

Metabolic activity was evaluated using colorimetric measurements with 3-[4,5-dimethylthiazol-2-yl]-2,5 diphenyl tetrazolium bromide solution (MTT, Sigma Aldrich) reagent. Cells were seeded into 96-well plates at 1 ×  10^4^ cells/well density in complete medium, grown overnight, and then incubated in the presence of GaAFP (50, 100, 200, 400 µg/mL) for 24 h. After this, 25 µ L of MTT (5 mg/mL) was added, and cells were incubated for 3 h at 37 °C. After this time, the medium was discarded, 100 µ L of dimethyl sulfoxide (DMSO) was added to each well, plates were shaken for 15 min, and absorbance was read in a microplate reader at 570 nm. Cell metabolic activity (cytotoxicity) was calculated as the percentage of the value obtained for cells incubated with compounds in comparison to control cells treated with the medium.

The level of NO production was determined in RAW 264.7 cells, which after attachment were treated with lipopolysaccharide (LPS, O55: B5 from *Escherichia coli*) (Millipore Sigma, Darmstadt, Germany) at 1 µ g/mL for 24 h. Then, 400 µg/mL AFP protein was added for additional 24 h. After cells’ treatment, the medium was collected, and the accumulation of NO metabolite in the cell culture supernatant was measured using Griess reagent (1% sulfanilamide and 0.1% naphthylethylenediamine dihydrochloride; Sigma Aldrich), where 50 µ L of the supernatant was mixed with 50 µ L of Griess reagent (40 mg/mL) in a 96-well plate. After incubation at room temperature and darkness for 10 min, the absorbance was measured at 540 nm. Cells treated with LPS without GaAFP were used as the positive control of the inflammatory response.

### Modelling of GaAFP structure

SWISS-MODEL [32] was used to create a model of GaAFP using homology modelling. Based on default settings and the query comprising the amino acid sequence of GaAFP (Uniprot ID I1E4B7), 17 templates were proposed, out of which the highest ranked crystal structure of LeIBP (Uniprot ID C7F6X3) deposited under Protein Data Bank (PDB) ID 3UYU [33] was chosen as a template. The report was inspected, and the generated model of GaAFP was visualized and compared to LeIBP in PyMOL software version 2.5.2. Blastp (at NCBI) alignment of modelled GaAFP and template LeIBP amino acid sequences was performed with the default parameters (expect threshold 0.05, word size 3, BLOSUM62 matrix with conditional compositional score matrix adjustment and gap costs: 11 for existence and 1 for extension).

### Statistical analysis

Calculations and plots were prepared using Microsoft 365 Excel (Microsoft Corporation, Redmond, WA, USA). Experimental values were reported as the means ±  SD. Statistical significance of the data was assessed by analysis of variance (*p* <  0.05) using the MiniTab 22 software (MiniTab Lt, Coventry, UK). For significant differences, the Tukey method of multiple comparisons was performed.

## Results and discussion

### Psychrophilic yeast identification

**Fig 1:**
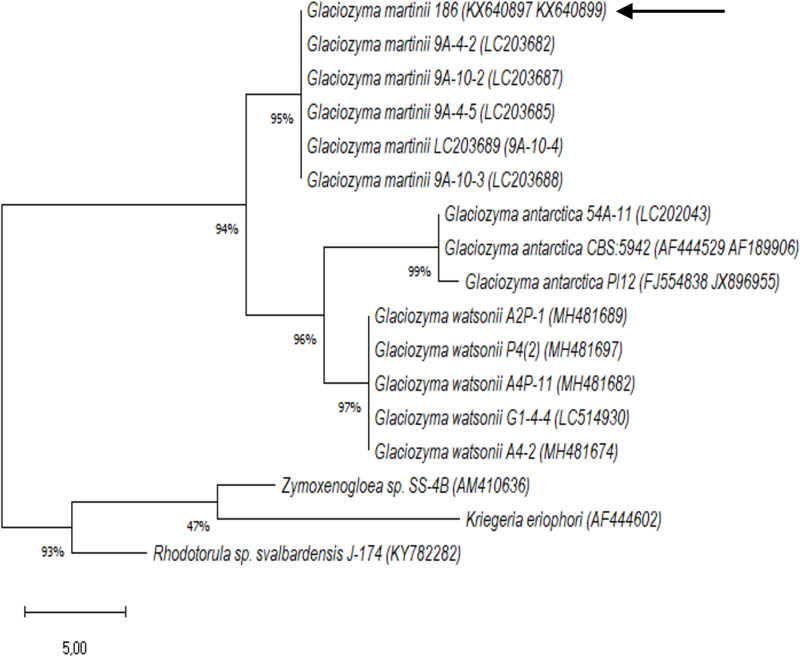
Phylogenetic similarity of *Glaciozyma martinii* 186 to other yeast strains. Analysis was made based on the sequences of the ITS1-5.8S rDNA-ITS2 region and the D1/D2 26S rDNA domains. The black arrow indicates *Glaciozyma martinii* 186 sequences; other sequences are described by species name and accession number in the GenBank database. Analysis was performed using MEGA 11 software. The topology of the phylogenetic tree was rooted with the *Rhodotorulla svalbardensis* J-174.

Based on the results of sequencing the D1/D2 regions of the 26S rDNA domain and the ITS1–5.8S–ITS2 region, the psychrophilic yeast strain number 186 from the collection of the Institute of Molecular and Industrial Biotechnology was classified to the species *Glaciozyma martinii* with over 99.6% agreement in BlastN (S4 Table). Among the selected species with the highest percentage of identity to the tested sequences (ITS1–5.8S–ITS2 and D1/D2 regions), the strains of *Glaciozyma antarctica* (~94%) and *Glaciozyma watsonii* (~94%) were also found. Fig 1 presents the phylogenetic relationship between the yeast closely related to the newly reported psychrophile *Glaciozyma martinii* 186. Until 2011, the species *Glaciozyma antarctica* was classified as *Leucosporidium* yeast. However, Turchetti et al. [34], after analyzing the biodiversity of Antarctica, Greenland, and Italian glaciers, discovered several previously undescribed psychrophilic yeast species unable to grow at temperatures above 20°C [34]. Analysis of the ITS1–5.8S–ITS2 and D1/D2 regions showed that the studied strains belong to unknown species related to *Leucosporidium antarcticum*. Two species were identified among the tested samples, and a new genus—*Glaciozyma*—was proposed. Additionally, a name change was proposed for *Leucosporidium antarcticum* to *Glaciozyma antarctica*, because, together with the recently described species (*Glaciozyma martinii* sp. and *Glaciozyma watsonii* sp.), they form a monophyletic clade and a well-separated lineage within the class Microbotryomycetes (Pucciniomycotina, Basidiomycota). Among the above-mentioned yeast species, only the genome of *Glaciozyma antarctica* PI12 has been sequenced to date. Unfortunately, there are also no reports on the yeast *Glaciozyma watsonii* and its ability to produce AFPs.

### Native GmAFP from *Glaciozyma martinii* 186—isolation and purification

**Fig 2:**
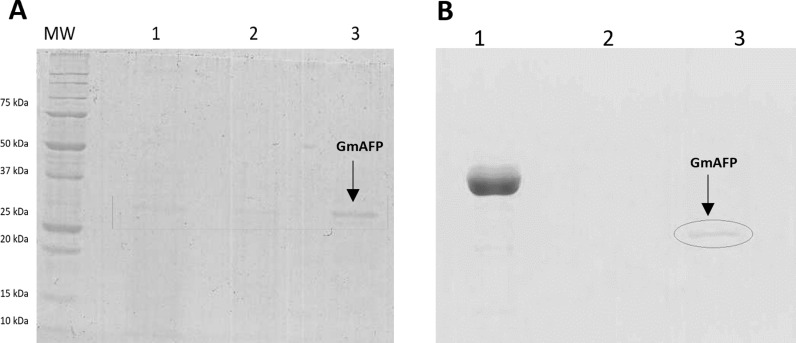
SDS-PAGE analysis of native GmAFP purification. A–purification steps: MW–molecular weight marker, 1–post-culture liquid, 2–proteins not bound to ice affinity chromatography, 3–fraction bound to ice; lanes with GmAFP are indicated by a grey rectangle; B–glycosylation staining using Periodic Acid-Schiff method: 1–positive control (horseradish peroxidase), 2–negative control (soybean trypsin inhibitor), 3–purified GmAFP from *Glaciozyma martinii* 186 indicated by a grey circle. Uncropped gels available at S3 Fig.

*Glaciozyma martinii* 186 yeast is an obligate psychrophile with an optimal growth temperature of 15°C. When cultured on a solid NB medium, it forms compact, clumpy, and matted aggregates of irregular shape (S2 Fig). Microscopic observation revealed the oval-shaped cells (S2 Fig). After 14 days of culture at 4°C in a liquid NB, the protein production yield was 30 mg/L from 2L of post-culture medium. The post-culture medium was concentrated and purified using ice affinity chromatography, which confirmed the presence of the native extracellular ~ 27 kDa AFP from *Glaciozyma martinii* 186, referred to as GmAFP (Fig 2A). The analysis of the presence of sugar residues in the protein molecule confirmed the glycosylation of GmAFP from *Glaciozyma martinii* 186 (Fig 2B).

*Glaciozyma martinii* 186 is the third yeast species whose AFP synthesis has been characterized. Scientific reports on ice-binding proteins of this origin are less common compared to those from other psychrophilic organisms. In 2010, Lee and colleagues [35] discovered an AFP, referred to as LeIBP (Uniprot ID C7F6X3), derived from the psychrophilic yeast *Glaciozyma* sp. AY30 (previously classified as *Leucosporidium*) isolated from an ice core sample from a freshwater pond in the Svalbard Archipelago, Norway [35]. This protein of approximately 25 kDa possesses both IRI and TH activities. LeIBP is currently the best-characterized yeast AFP, and its gene has been expressed in two expression systems: *Escherichia coli* and *Pichia pastoris* [36]. Additionally, the crystal structure of this protein was solved and deposited in the PDB under the accession numbers: 3UYU, 3UYV [33,37]. Another strain exhibiting antifreeze activity is *Glaciozyma antarctica* PI12 [7,38]. This yeast secretes two types of AFPs: Afp1, with a mass of approx. 18 kDa [7], and Afp4, with a mass of approx. 25 kDa [38]. Both molecules demonstrate TH (0.08°C) and IRI activities. Moreover, Afp4 shares over 93% sequence similarity with the LeIBP protein (from *Glaciozyma* sp. AY30). Apart from yeasts of the *Glaciozyma* genus, antifreeze activity has been demonstrated in yeasts species *Rhodotorulla glacialis*, *Leucosporidium creatinivorum*, *Candida parapsilosis*, and *Goffeauzyma gastrica* [10,39]. Unfortunately, proteins derived from these microorganisms have not yet been fully characterized.

Due to the low yield of GmAFP protein production by the native producer *Glaciozym amartinii* 186 and the lengthy culture time (14 days) at a low temperature (4°C), coupled with the low efficiency of the purification process, it was decided to conduct an expression of a homologous recombinant AFP. The only available genome of the closely related yeast was from *Glaciozyma antarctica* PI12 [40]. The genome of the yeast *Glaciozyma martinii* remained unresolved; hence, the *afp* gene from the native producer *Glaciozyma martinii* 186 coding for GmAFP could not have been isolated despite multiple trials. Finally, it was decided to opt for the large-scale production in the mesophilic host *Pichia pastoris* of Afp4 protein from *Glaciozyma antarctica* PI12, referred to as GaAFP (Uniprot ID I1E4B7).

### Vector construction, protein expression and purification of recombinant GaAFP from *Glaciozyma antarctica*

**Fig 3:**
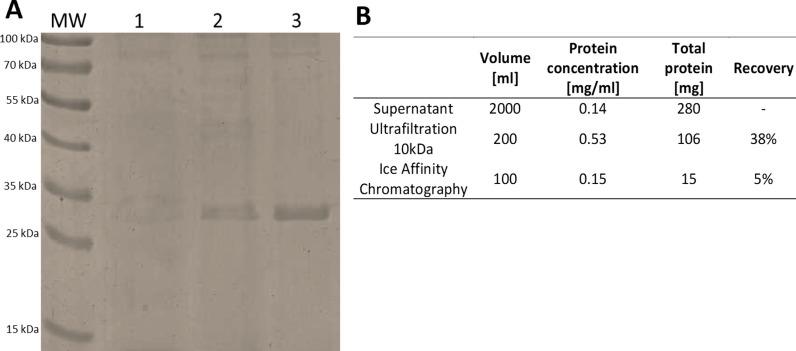
Purification steps of recombinant GaAFP. A–SDS-PAGE analysis, lanes: MW–molecular weight marker, 1–supernatant after 72 h expression at 28°C with 1% of methanol induction, 2–post-culture liquid concentrated using tangential flow filtration, 3–fraction bound to ice affinity chromatography; B–yields of purification after each step. Uncropped gel available at S7 Fig.

The synthetic sequence of the gene encoding for GaAFP protein (Afp4 from *Glaciozyma antarctica* PI12; sequences available in the S1 Table) was cloned into pPICZB plasmid, which was corroborated by 100% identity in sequencing. The presence of the gene in transformant colonies was confirmed by colony PCR. In order to increase the efficiency of GaAFP protein expression, optimization of the culture process of the chosen *Pichia pastoris* GS115 recombinant colony was carried out. In short, based on semiquantitative analysis by SDS-PAGE (S4 Fig), the most optimal conditions for GaAFP production was 28°C and induction of 1% methanol twice a day during 72 h of cultivation. The recombinant GaAFP consists of 261 amino acids and has a molecular weight of 26.57 kDa. It contains a signal peptide comprising the first 20 amino acids (S5 Fig), which enables the extracellular production of GaAFP. Additionally, the glycosylation site of the recombinant GaAFP was found based on the amino acid sequence analysis (S6 Fig). The obtained protein was is within the expected size (Fig 3A). The post-culture liquid, with a protein concentration of 140 mg/L, was subjected to centrifugation, concentration and purification on ice affinity chromatography.

After purification on the ice affinity chromatography, only a 5% protein recovery was obtained (Fig 3B). This method is dedicated to AFPs and allows for their separation from the rest of the extracellular proteins and other components of the culture medium. On the other hand, one of the disadvantages is the long duration time and dilution of the sample by the growing ice. However, using canonical techniques (e.g., gel filtration, ion exchange chromatography) to purify recombinant AFPs from large-scale fermentation process showed similar percent of protein recovery [36]. High specificity of the ice affinity chromatography method allowed for a great simplification of protein purification procedure.

## Microscopic analysis of the ice recrystallization inhibition (IRI)

**Fig 4:**
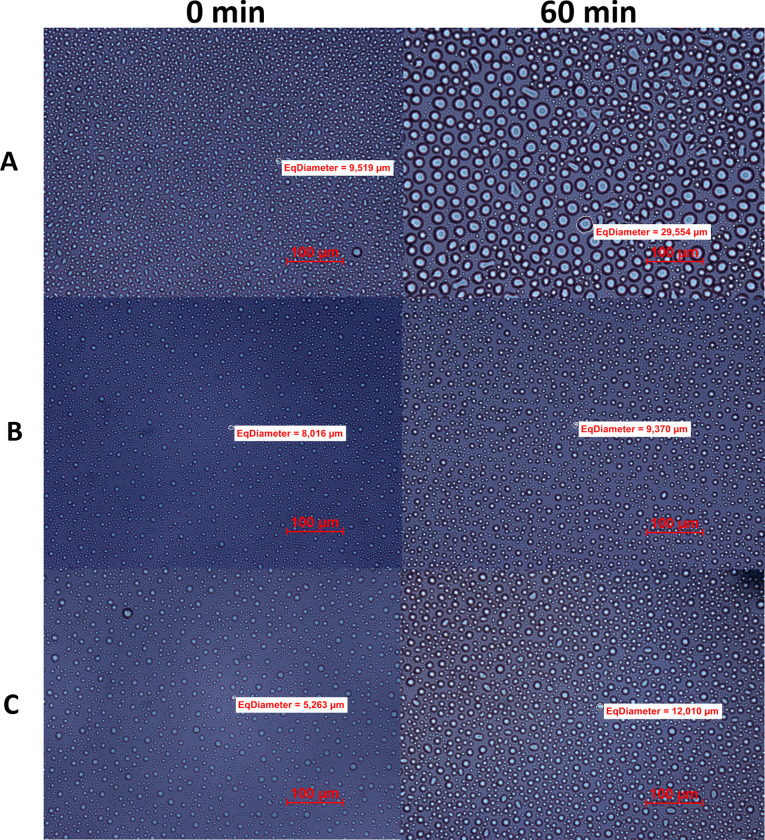
Microscope analysis of ice recrystallization inhibition (IRI). The following samples were tested during the analysis: native protein from *Glaciozyma martinii* (B–GmAFP), recombinant protein from *Glaciozyma antarctica* (C–GaAFP), compared to sucrose solution (A–negative) in time 0 and after 60 min of incubation at –8°C at the cooling stage. Example of equivalent diameter marked on the figures (rest parameters: minimal, maximal and average ±  SD size crystals available in S3 Table): Negative sample: 0 min–9.519 µm, 60 min–29.554 µm; GmAFP: 0 min–8.016 µm, 60 min–9.370 µm; GaAFP: 0 min–5.263 µm, 60 min–12.010 µm. Scale bar 100 µm.

**Fig 5:**
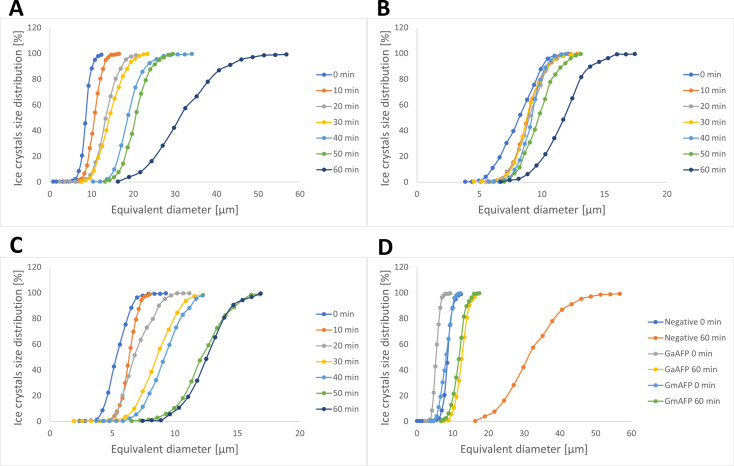
Distribution of ice crystal size during ice recrystallization inhibition (IRI) assay at different time intervals. A–negative sample (sucrose); B–native protein from *Glaciozyma martinii* 186 (GmAFP); C–recombinant protein from *Glaciozyma antarctica* (GaAFP); D–comparison of all samples during 0 and 60 min of incubation. The data used to draw the graphs are presented in S3 Table.

Microscopic analysis of IRI with sucrose is one of the t commonly used methods to determine ice growth rates. High concentration of sugar in the sample allows for observation of ice crystals under the microscope, as it impedes water flow between them. Fig 4 illustrates the ice growth of all tested samples at 0 and 60 min after placing under the microscope with a cooling stage. In the negative control sample, which is the sucrose solution, after 60 min of incubation at ‒8°C, considerably sized ice crystals were observed (mean diameter 32.58 ± 3.75 µm, S3 Table), indicating a substantial ice recrystallization phenomenon. However, when observing AFP-containing solutions, both native (GmAFP) and recombinant (GaAFP) under the same conditions, recrystallization was inhibited. The average diameter of the ice crystals after 60 min was respectively 11.69 ± 1.85 µm and 12.53 ± 1.73 µm (S3 Table). The average size of ice crystals after 60 min of incubation at ‒8°C for the negative control was more than two times greater than that of the test samples containing the AFPs. Additionally, there is a clear trend of increasing ice crystal size for the negative sample at all measured points (Fig 5A), reaching a maximum crystal size of 55 µm, whereas for the test samples, this increase is gradual, with the largest crystal recorded being approximately 18 µm in diameter (Fig 5B-C), three times smaller than that of the negative sample. Based on the graphs shown in the Fig 5D, the value of the ice crystal diameter at 50% of the cumulative distribution of the sample (X_50_ diameter) was calculated. For each of the tested samples, this parameter fluctuated within the average size of the ice crystals (Negative—32.84 µm; GmAFP—11.79 µm; GaAFP—12.44 µm).

In 2014, Gaukel et al. [41] conducted a similar experiment, describing the effects of different AFPs on IRI [41]. Tested separately and in combinations, AFPs reduced the size of growing ice crystals after several hundred hours of incubation at ‒8°C by three to six times compared to a negative control, where the test solution contained 49% sucrose. Similar observations regarding ice crystal growth dynamics were reported by Rahman et al. in 2019 [6]. During their study, ice crystal size was compared for a 40% sucrose solution and different concentrations of AFP I at 5min intervals. The sucrose solution exhibited considerable crystal growth, with the measured cubic radius being twice as large after 25 min of incubation at ‒6°C compared to the AFP I sample with the highest measured concentration of 10 µ M. Another scientific report focused on genome sequence analysis of the yeast *Glaciozyma antarctica* PI12, revealing the presence of nine genes encoding AFPs [40]. Afp 3, 4, 5, 7, and 9 genes were successfully cloned for expression in *Escherichia coli*, but unfortunately, the recombinant proteins were obtained only in inclusion bodies. These proteins were purified after refolding, and their effect on the shape of the ice crystals formed was examined. It was observed that these AFPs exhibited low TH activity (0.03‒0.08°C) and high IRI activity, as measured by the combination of microscopic analysis with a sucrose solution [40]. These results confirm that protein expression in a heterologous host, such as the yeast *Pichia pastoris* or *Escherichia coli*, enables the production of proteins that are as functional as their native counterparts.

### Cell structure of vegetables and fruit

**Fig 6:**
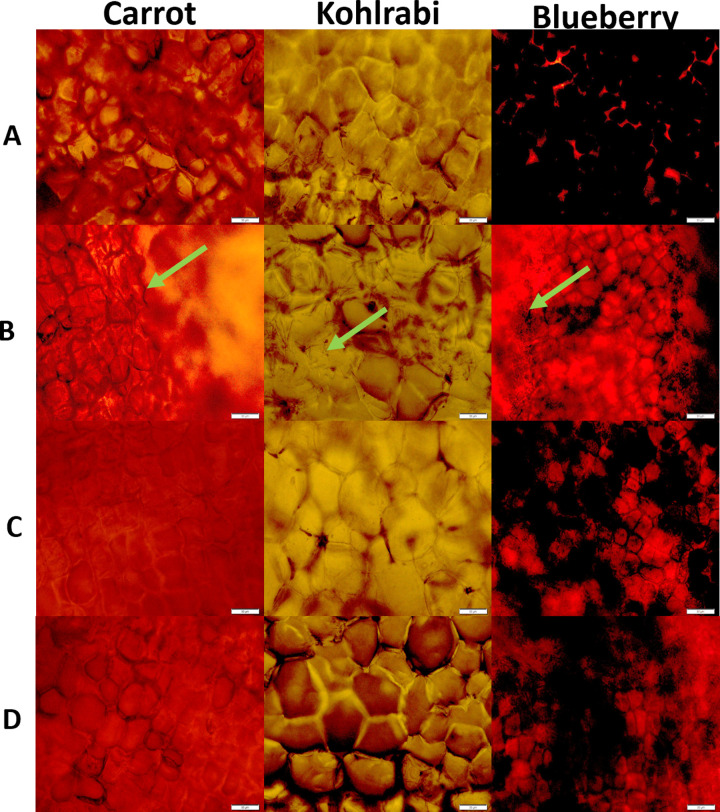
Structure analysis of vegetables and fruits after 16 h of freezing at –20°C. A–freshly cut vegetables or fruits. B–sample without the addition of a cryoprotectant. The following solution were tested during analysis: D–solution of GaAFP (0.1 mg/mL) or C–glycerol (0.5 M). Green arrows indicate the damaged area after freezing without cryoprotectant. White–scale bar–50 µm.

The experiment was designed to show the cryoprotective role of recombinant GaAFP during freezing of chosen hard vegetables (carrot, kohlrabi) and soft fruits (blueberry). Pre-soaking with 0.1 mg/mL GaAFP for 16 h prior to freezing with produced results comparable to glycerol, a widely utilized CPA. Microscopic analysis of cells showed intact structures without visible ruptures, in contrast to negative samples (Fig 6). Furthermore, cells retained pigment, as demonstrated by blueberry samples. Similar observations were made for strawberries frozen with fish AFP I [42]. Thawed strawberries treated with 0.02 g/L AFP I exhibited reduced damage to cell microstructures and closely resembled fresh strawberries under microscopic observation. Additional reports suggested that AFPs from Antarctic bacteria with TH activity (ranging from 0.17 to 0.5°C), combined with a commercial type III-AFP as a positive control, safeguarded cucumber and zucchini cells integrity during freezing-thawing cycles [43].

### Drip loss analysis

The primary challenge encountered by the food industry when freezing fresh fruits and vegetables is the drip loss [44]. Freezing damages cells, causing substantial water leakage along with other substances during thawing, resulting in diminished food quality. The experiment aimed to demonstrate the impact of GaAFP on the extent of water leakage, depending on pre-soaking time and the type of food.

**Fig 7:**
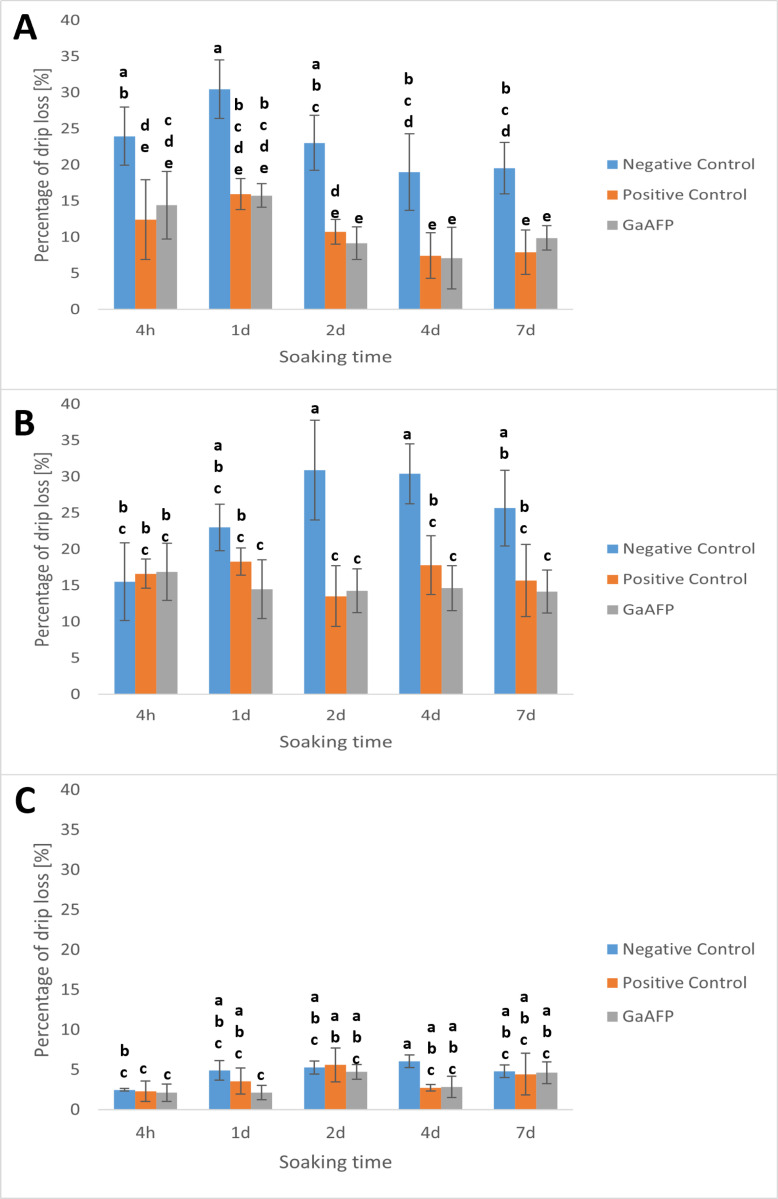
Drip loss analysis of vegetables or fruits. Tested samples were frozen with 0.1 mg/mL GaAFP: A–carrot, B–kohlabri, C–blueberries. Positive control: 0.5 M glycerol, negative control: no cryoprotectant. The data are presented as a means ±  SD from 5 replicates. The means that are marked with different letters are significantly different (p <  0.05).

The effect of recombinant GaAFP varied depending on the sample. For carrots, the protective effect was evident; GaAFP protein exhibited a similar effect to glycerol, and the results markedly differed from the negative control (Fig 7A). Moreover, with an increased soaking time, the effect improved. Kong et al. [45] demonstrated a similar effect on carrot samples by freezing them with three different chemically synthesized antifreeze peptides [45]. These synthetic peptides were found to modify the morphology and size of ice crystals at a minimum concentration of 0.01 M. Furthermore, it was shown that they can function as cryoprotectants in food. Pre-soaking samples in solutions of synthetic AFP peptides (0.1 mg/mL) before freezing helped maintain the texture of frozen carrots akin to fresh carrots, reduced drip loss, minimized structural damage from freezing, and preserved the unique carrot flavor [45].

Similar results were obtained for kohlrabi (Fig 7B). However, the results for soaking within 4 h were in the same level, which may indicate that this is a too short soaking time for this vegetable. In contrast, most of the results for blueberries were similar for each sample (Fig 7C). Blueberries, being a soft fruit, were frozen whole. It is possible that the peel prevents water leakage during the thawing process for negative samples, and for positive samples (glycerol and GaAFP), it may act as a barrier against penetration.

This is the first time the cryoprotective effects of yeast-derived AFPs have been demonstrated in the food industry (Fig 6 and 7). Most reports related to food cryoprotection described the AFPs of plant, animal (insect, fish) or bacterial origin [42,43,46,47]. One of the reports used extracts of cold-acclimated leaves of *Drimys angustifolia* in the storage of carambola fruit [48]. In addition to a reduction in drip loss, the samples were further characterized by increased firmness after thawing. Another study presented cryoprotection of vegetables (courgette, carrot, cucumber and onion) were tested with AFP from the insect *Tenebrio molitor* was used at a concentration of 1 mg/mL [46]. The results for vegetables were similar and demonstrated that the addition of AFP protected the cell wall structures and allowed the products to retain their structure during storage.

To date, the only type III AFPs derived from psychrophilic fish have been approved for consumption and permitted in the use as ice cream additive in 2008 by the European Food Safety Authority in Europe [49] and in 2013 by the Food and Drug Administration in the USA [50]. The inclusion of AFP in ice cream mixes ensures the stability of their texture and organoleptic properties, which are highly valued by consumers, even during slight temperature fluctuations during the storage of the final product [51].

Microorganisms serve as an excellent source of AFPs due to their ease of cultivation. They require only a few days of cultivation at reduced temperatures to produce AFPs, which is unattainable for plants and fish. Furthermore, the ability to cultivate organisms like yeast in fermenters allows for complete automation of the process [36]. Additionally, by designing a suitable expression construct for a mesophilic host, the production of these proteins at a large scale can be shortened without the necessity of lowering the temperature.

### Viability test of frozen *Saccharomyces cerevisiae* yeast

**Fig 8:**
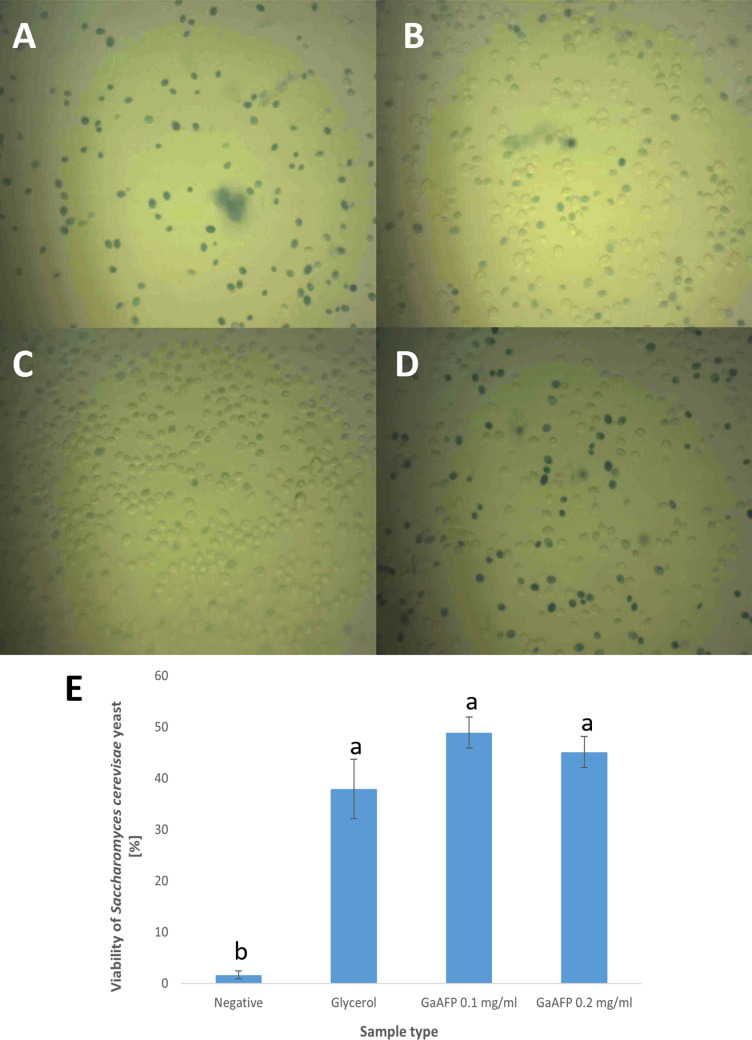
Viability of frozen *Saccharomyces cerevisiae* yeast after 35 days of freezing at ‒20°C. A–D: microscopic analysis: A–negative sample (yeast frozen without cryoprotectant), B–positive sample (0.5 M glycerol), C–0.1 mg/mL GaAFP protein, D–0.2 mg/mL GaAFP protein. Viable cells are transparent, whereas dead cells are visible as blue after staining with methylene blue (magnification × 400); E–percent of viability of frozen *Saccharomyces cerevisiae* yeast. The data are presented as mean of three replicates with ± SD. The means that are marked with different letters are significantly different (p <  0.05).

The addition of 0.1 mg/mL and 0.2 mg/mL recombinant GaAFP protein positively influenced yeast cell survival after 35 days of freezing (Fig 8). Both protein concentrations had a similar effect, and the % of viable yeast cells after the freezing process were not significantly different. GaAFP protein exhibited a slightly superior effect of survival rate (0.1 mg/mL–48.93% and 0.2 mg/mL–45.13%) compared to the standard CPA—glycerol (37.93%), which is commonly used in microbiology. The absence of a cryoprotectant resulted in the majority (98.33%) of frozen yeast cells failing to survive the freezing-thawing process. The baker’s yeast *Saccharomyces cerevisiae* was selected due to its wide application in the food industry, including baking, dairy production, and brewing. Another widely used lactic acid bacteria in the food industry, *Streptococcus thermophilus*, was also subjected to a similar test. Chen et al. used 0.1 mg/mL (the same concentration as in our work) of a novel recombinant antifreeze peptide derived from snow flea (rsfAFP) [52]. The observations were similar, the rsfAFP improved the survival rate of lactic acid bacteria (93.21% survival rate) compared to typical cryoprotectants (15% glycerol–79.80% survival rate), after freezing at − 20°C for 24 h and 2 freezing-thawing cycles [52].

Most of the scientific reports using AFPs as cryoprotectants concern the storage of human or animal cells or tissues. The most investigated cells are sperm [53,54], oocyte [55–57], embryo [58], red blood cells [59] and cells line (e.g., HeLa, NIH/3T3, MC3T3-E1 or CHO-K1) [60] or mouse ovarian tissues [61,62]. The cryoprotective effect depend of type of antifreeze protein, its concentration, storage time and storage condition. Cryoprotective effect on the microorganisms was also demonstrated when marine diatoms *Phaeodactylum tricornutum* were frozen with LeIBP from the Arctic yeast *Leucosporidum* sp. AY30 [63]. *Phaeodactylum tricornutum* is one of the model organisms for diatoms. Its cells were subjected to two types of freezing: fast and two-stage freezing with the addition of classically used CPAs (dimethyl sulfoxide, glycerol, ethylene glycol; EG, and polyethylene glycol; PEG), and with or without a supplement of LeIBP. Two-step freezing resulted in substantially increased cell survival, which was statistically the same as for unfrozen native cells with addition of 0.1 mg/mL LeIBP in 10% EG or 10% PEG at 11 day of post-thaw culture. Furthermore, a considerable increase in chlorophyll concentration was observed with the addition of LeIBP and all the above-mentioned CPAs. In addition, microscopic analysis showed no change in cell morphology [63].

### Effect of GaAFP on the metabolic activity of cells and nitic oxide secretion

**Fig 9:**
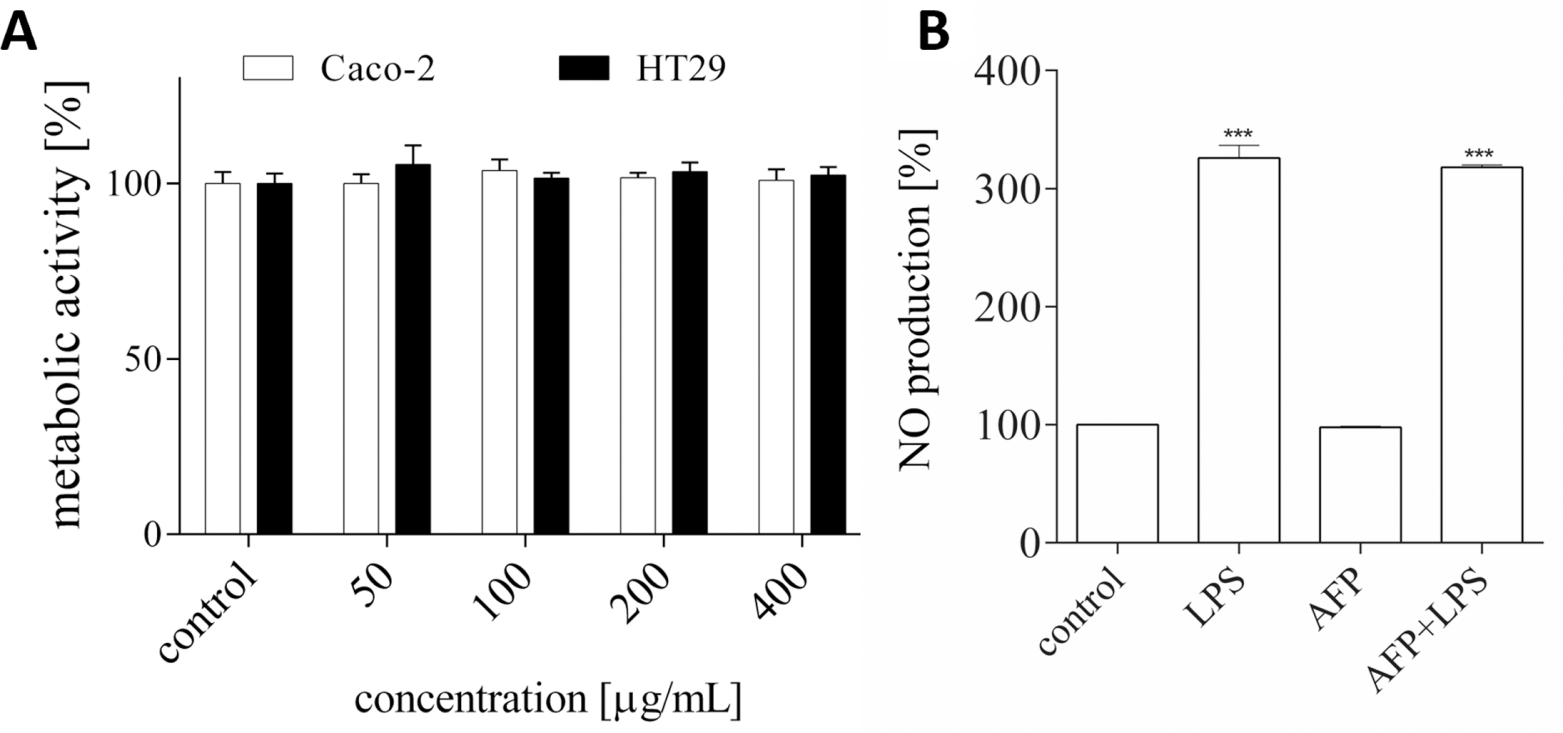
Effect of GaAFP on metabolic activity of cells and nitic oxide secretion. A–the influence of GaAFP protein on Caco-2 and HT-29 cells’ metabolic activity after their 24-h incubation at 50–400 µ g/mL concentration determined with MTT assay. B–effect of 400 µ g/mL GaAFP on nitric oxide (NO) production in LPS-stimulated RAW 264.7 cells determined with Griess reagent. Control cells were only exposed to the vehicle. The values in each column represent the mean ±  SEM, **n** ≥  4. Significance differences were calculated against LPS-treated cells with *** ****p**** ≤  0.001.

Since the AFP proteins are intended to be used in the food industry, we aimed to evaluate their potential cytotoxic effect with an in vitro study involving cells originating from the digestive tract. The biological activity of GaAFP has been examined with human colon adenocarcinoma Caco-2 and HT-29 cell lines, which are routinely used as a cellular model of intestine barrier in bioavailability studies. As shown in Fig 9A, after cells’ incubation for 24 h in the presence of AFP at the range of 0–400 µ g/mL, no decrease of the metabolic activity was observed in both types of cell lines. Therefore, we decided to determine the effect of GaAFP protein on the secretion of nitric oxide (NO), which is one of the main mediators of the chronic inflammatory process. As a cell model we used mice macrophages RAW 264.7 which are frequently involved in studies carrying out in vitro screening for immunomodulators. Due to lack of the negative impact on cells metabolic activity, we determined the GaAFP effect at the highest studied concentration (400 µ g/mL). As demonstrated in Fig 9B in LPS-induced RAW 246.7, the level of NO release significantly increased, achieving almost 300% in comparison to unstimulated control. GaAFP had no effect on NO secretion; in cells stimulated with LPS or without the stimulation, the level of NO was comparable to that of the control cells. Our results are in accordance with the data presented by Tran-Guzman et al., 2022, where after incubation of different types of cells (C18-4, MA-10, RAW 264.7, HUH7), slight cytotoxicity was observed for concentrations higher than 500 µ g/mL after 48 h incubation [64]. In our case, we selected a shorter time of incubation (24 h), which resembles more appropriately the time of the duration of food’s passage through the digestive system. Based on these preliminary results, we can suspect that AFP protein can be used as a safe additive to the food, however, this statement requires more detailed in vivo studies.

### Modelling of GaAFP structure

**Fig 10:**
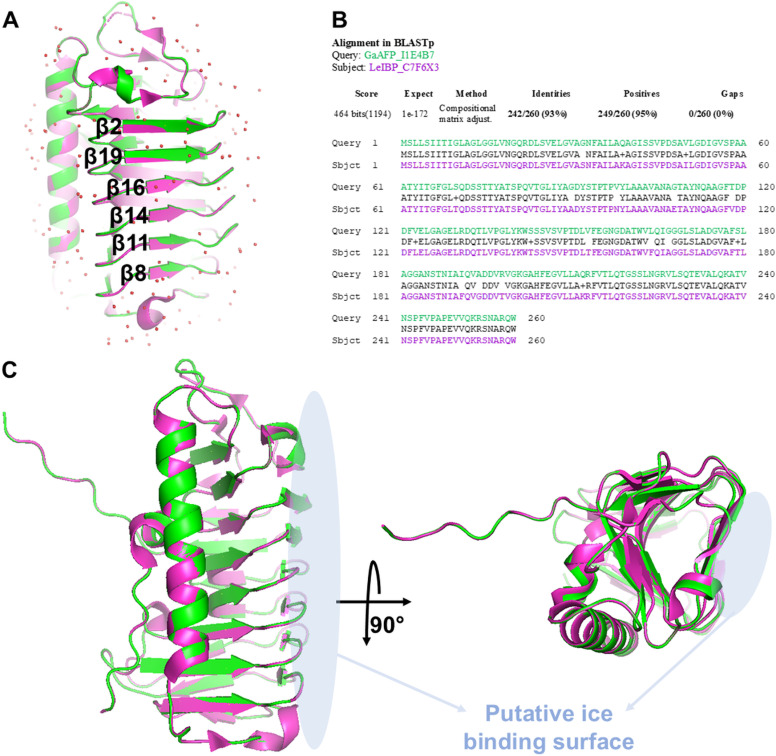
Model of GaAFP structure. A–Model of GaAFP structure (green cartoon) based on the template of chain A of homologous microbial LeIBP (magenta cartoon, waters visualized as red spheres); β-strands engaged in the putative ice-binding surface are labelled; B–Blastp alignment of amino acid sequences of GaAFP (Uniprot ID I1E4B7, presented in green) and the template sequence of LeIBP (Uniprot ID C7F6X3, presented in magenta); C–Comparison of GaAFP model and LeIBP X-ray structure in the context of putative ice-binding surface (marked in light blue).

To investigate the putative ice-binding mechanism, homology modelling was performed in SWISS-MODEL [32], which yielded a 3D model of the structure of GaAFP based on the template of homologous microbial LeIBP (PDB ID 3UYU). The overall monomeric structure of GaAFP adopts a right-handed helical fold. Chains A of the model of GaAFP and the X-ray structure of LeIBP (PDB ID 3UYU) aligned perfectly with a very low RMSD of 0.066 Å (Fig 10A). This is not surprising since the Blastp alignment of these two protein sequences results in 93% identities and 95% positives (Fig 10B). The shape of the putative ice-binding surface of GaAFP (Fig 10C) is similar to that postulated by Lee et al., 2012 in the analysis of the experimental structure of LeIBP [33,37]. Six β-strands engaged in the putative ice-binding site overlap with those of a template and create a flat, slightly concave surface. However, it should be noted that the final ice-binding mechanism could only be defined after determining the X-ray structure of the GaAFP, which constitutes the further step of our research.

### Limitations of the study

The Institute of Molecular and Industrial Biotechnology has a large collection of psychrophilic and psychrotrophic microorganisms. For many years, we have gradually published reports on new metabolites, including psychrozymes derived from low-temperature organisms [65–68]. Furthermore, we have experience in the production of recombinant proteins in bacterial or yeast expression systems. One of the intriguing subjects of our research comprises the AFP proteins, which, due to their properties, translate into molecules with a high-application potential, e.g., in the food industry. The psychrophilic yeast *Glaciozyma martinii* 186 was found to be a new source of these proteins, which is especially interesting in the context of the scarcity of information about the AFP-producing microorganisms.

Our research has a certain number of limitations. Firstly, we were not able to demonstrate the applicability of the native AFP from *Glaciozyma martinii* 186 (GmAFP) in the food industry, mainly due to the low concentration of the native protein and the long culture time of the producer. The other limitation is connected with the purification of AFP, which includes ice affinity chromatography performed with the laboratory-assembled equipment. However, compared to animal and plant AFPs, microbial proteins could be produced at lower costs due to their rapid growth coupled with simple and inexpensive nutrient sources. Additionally, difficulties were encountered in isolation of the gene encoding for GmAFP, which prevented its expression in a heterologous host and hindered the increase in the production efficiency. We plan to conduct the sequencing of the genome of *Glaciozyma martinii* 186 and perform bioinformatic analysis to identify the GmAFP gene in the near future. To combat the low yields of GmAFP secretion, we have opted for the heterologous expression of the homologous protein from *Glaciozyma antarctica* PI12, GaAFP, in the mesophilic host *Pichia pastoris*. Unfortunately, no commercial eucaryotic systems are dedicated to the expression of psychrozymes or other metabolites from cold-adapted microorganisms. Even though the amounts of the recombinant GaAFP were satisfactory to conduct the planned tests, the yield remained a limiting factor for large-scale production and is still a subject of optimization, mainly through the changes in the purification procedure.

## Future Outlook

In this study, the novel native GmAFP from the newly isolated psychrophilic yeast *Glaciozyma martinii* 186 was purified and characterized. Its IRI activity was confirmed through microscopic visualization. Due to low expression and purification yields, along with the extended cultivation time of the native strain, it was decided to synthesize the *afp* gene from a closely related yeast species, *Glaciozyma antarctica* PI12. Expression of recombinant GaAFP in the mesophilic host, *Pichia pastoris*, resulted in nearly a five-fold increase in total protein production, while reducing the cultivation time by almost three-fold. GaAFP functions similarly to the commonly used cryoprotectant—glycerol. Other common CPAs, such as dimethyl sulphoxide, polyvinyl alcohol, PEG, EG, trehalose or sucrose, can be toxic or produce adverse effects when used in high concentrations. On contrary, AFPs are non-toxic and do not alter the taste or physical properties of solutions, hence, become ideal candidates for modern CPAs. They have an unlimited potential for application not only in the food industry, but also in the cryopreservation of cells, tissues or organs.

## Supporting information

S1 Fig Ice affinity chromatography for AFP protein purification. Ice affinity chromatography system: A–mechanism of action this method for purification of antifreeze proteins, B–the apparatus made in laboratory. Using affinity chromatography, besides selectively purifying the AFP protein, we effectively separated the coloured components of the culture medium. Briefly, a cooling finger with flowing ethylene glycol was immersed in distilled water containing small ice crystals to create a thin ice layer for the AFP proteins to adhere to. Subsequently, a pre-chilled solution of post-culture medium was introduced. The cold finger, positioned approximately 20 mm from the bottom, was continuously rotated using a magnetic stir bar. The purification process took place overnight, maintaining the temperature of the cooling solution around −2°C, resulting in the freezing of approximately 50% of the initial solution. After the purification process, the ice formed was washed with distilled water and placed in a clean beaker. Elution was performed by thawing the cooling finger - the temperature of the ethylene glycol in the cooling system was raised to 2°C.
(TIF)

S2 Fig **The morphology of *Glaciozyma martinii* 186.** Yeast grown on A–Nutrient Broth Agar (NBA) at 6°C for 14 days. B–*Glaciozyma martinii* 186 colony unit growing on NBA observed under a light microscope (40 × magnification) C–*Glaciozyma martinii* 186 cells after 7 days growing on liquid NB with 180 rpm shaking observed under a light microscope (400 × magnification).
(TIF)

S3 Fig Full-length gels from the Figure 2a-b. A–SDS-PAGE analysis of native GmAFP purification–purification steps; B–glycosylation staining using periodic acid-Schiff method.
(TIF)

S4 Fig Analysis of GaAFP expression conditions based on SDS-PAGE. The optimization of AFP protein expression involved culturing *Pichia pastoris* GS115 in BMMY medium with varying final concentrations of methanol and different cultivation temperatures. Two culture times were considered: lanes 1–4 for a 48-hour cultivation and lanes 5–8 for a 72-hour cultivation. The conditions were as follows: 1–20°C, 0.5% methanol; 2–20°C, 1% methanol; 3–28°C, 0.5% methanol; 4–28°C, 1% methanol; 5–20°C, 0.5% methanol; 6–20°C, 1% methanol; 7–28°C, 0.5% methanol; 8–28°C, 1% methanol.
(TIF)

S5 Fig Signal peptide prediction in GaAFP protein. The SignalIP 6.0 bioinformatics tool confirmed the presence of a signal peptide enabling protein secretion in the first 20 amino acids of the expressed GaAFP protein with a probability of 0.999. Additionally, a cleavage site (CS) between the 20th and 21st amino acids was identified.
(TIF)

S6 Fig Predicted glycosylation site based on amino acids sequence GaAFP. The likely site of glycosylation in GaAFP is asparagine at position 185.
(TIF)

S7 Fig Full-length gel of Figure 3A. SDS-PAGE analysis, lanes: 1–supernatant after 72 h expression at 28°C with 1% of methanol induction, 2–post-culture liquid concentrated using tangential flow filtration, 3–fraction after ice affinity chromatography.
(TIF)

S1 Table Nucleotide and protein sequences of GaAFP. (PDF)

S2 Table List of primers using in cloning *afp* gene from *Glaciozyma antarctica.* (PDF)

S3 Table Comparison of ice crystal distribution in all tested samples and after different storage times. (PDF)

S4 Table Results of the sequence analysis (ITS region and D1/D2 domain) of closely related yeast species to *Glaciozyma martinii* 186 using BlastN. Results with the highest percentage of identity. Among the selected species with the highest percentage of identity to the tested sequence (ITS region and D1/D2 domain) were: *Glaciozyma martinii*, *Glaciozyma antarctica*, and *Glaciozyma watsonii*. *Glaciozyma martinii* exhibited the highest percentage of identity (over 99%), with sequence coverage for the selected phylogenetic marker close to 100% and minimal gaps. *Glaciozyma antarctica* displayed a lower percentage of identity at approximately 94% and a higher content of gaps (2%–3%) with coverage close to 100%. *Glaciozyma watsonii* showed a similar situation to *Glaciozyma antarctic*a. *Phenoliferia himalayensis* exhibited a much lower identity at 89.39% with nearly 100% coverage. The sequence identity of *Rhodotorula svalbardensis* was at 82.89% with a sequence coverage value of 85%. *Zymoxenogloea eriophori* demonstrated the lowest identity with 100% sequence coverage. From the listed sequences, those suitable for phylogenetic analysis using the MEGA11 software were selected.
(PDF)
