# Supplementary figures and images for: Antifreeze proteins produced by Antarctic yeast from the genus Glaciozyma as cryoprotectants in food storage
Source: PLoS One. 2025 Mar 6;20(3):e0318459. doi: 10.1371/journal.pone.0318459 (PMC11884722; doi:10.1371/journal.pone.0318459)

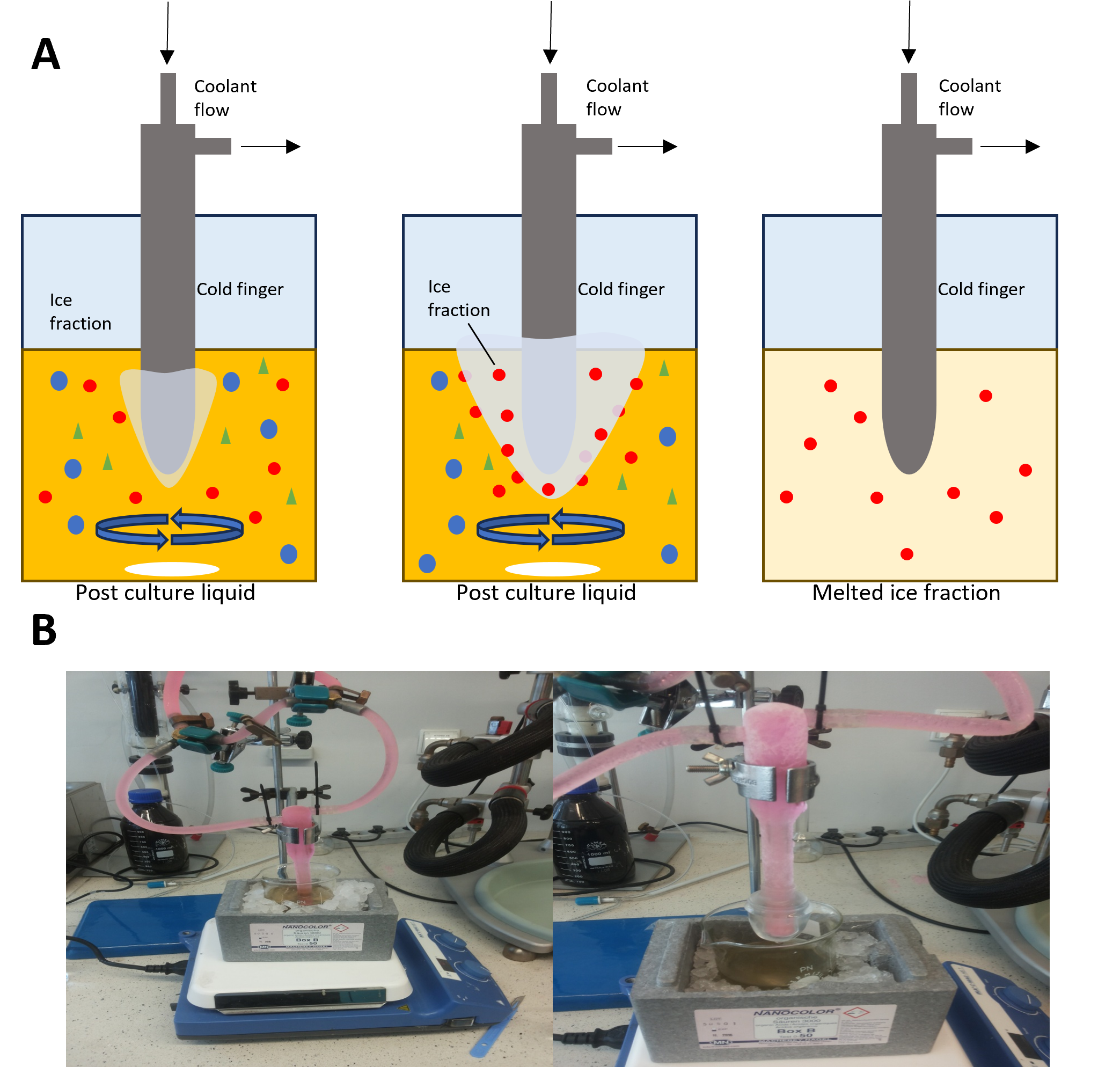

Supplement: S1 Fig — Ice affinity chromatography system: A–mechanism of action this method for purification of antifreeze proteins, B–the apparatus made in laboratory. Using affinity chromatography, besides selectively purifying the AFP protein, we effectively separated the coloured components of the culture medium. Briefly, a cooling finger with flowing ethylene glycol was immersed in distilled water containing small ice crystals to create a thin ice layer for the AFP proteins to adhere to. Subsequently, a pre-chilled solution of post-culture medium was introduced. The cold finger, positioned approximately 20 mm from the bottom, was continuously rotated using a magnetic stir bar. The purification process took place overnight, maintaining the temperature of the cooling solution around −2°C, resulting in the freezing of approximately 50% of the initial solution. After the purification process, the ice formed was washed with distilled water and placed in a clean beaker. Elution was performed by thawing the cooling finger - the temperature of the ethylene glycol in the cooling system was raised to 2°C. (TIF) [file pone.0318459.s001.tif]

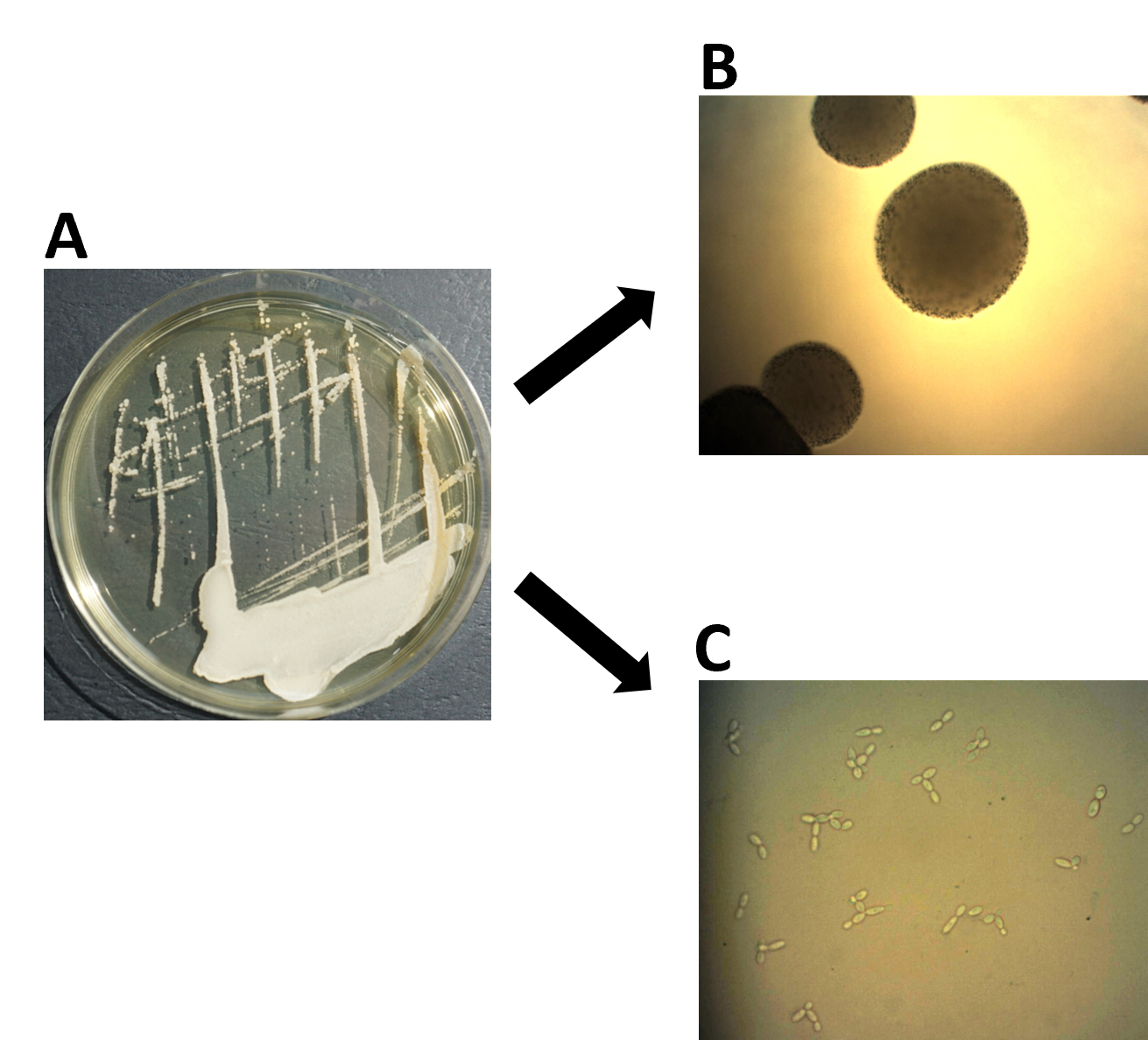

Supplement: S2 Fig — The morphology of Glaciozyma martinii 186. Yeast grown on A–Nutrient Broth Agar (NBA) at 6°C for 14 days. B–Glaciozyma martinii 186 colony unit growing on NBA observed under a light microscope (40 × magnification) C–Glaciozyma martinii 186 cells after 7 days growing on liquid NB with 180 rpm shaking observed under a light microscope (400 × magnification). (TIF) [file pone.0318459.s002.tif]

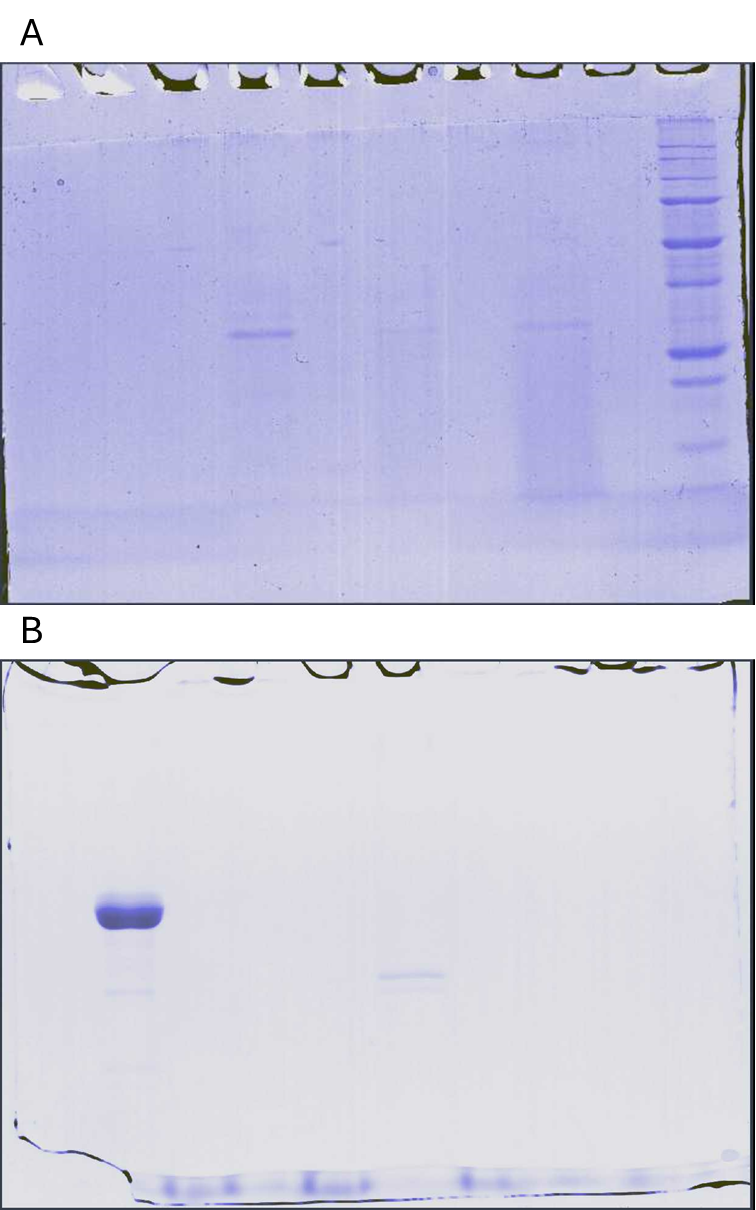

Supplement: S3 Fig — A–SDS-PAGE analysis of native GmAFP purification–purification steps; B–glycosylation staining using periodic acid-Schiff method. (TIF) [file pone.0318459.s003.tif]

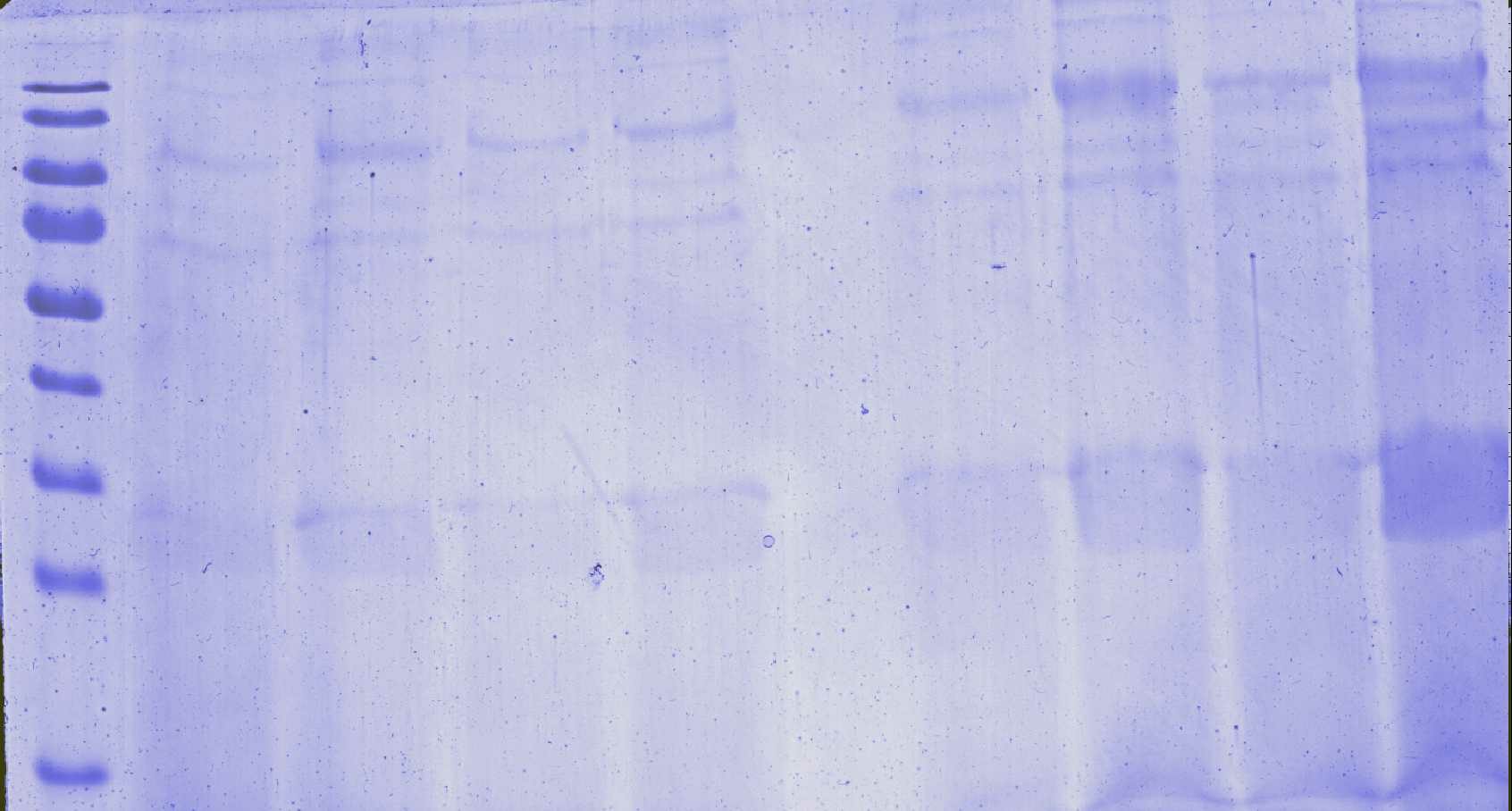

Supplement: S4 Fig — The optimization of AFP protein expression involved culturing Pichia pastoris GS115 in BMMY medium with varying final concentrations of methanol and different cultivation temperatures. Two culture times were considered: lanes 1–4 for a 48-hour cultivation and lanes 5–8 for a 72-hour cultivation. The conditions were as follows: 1–20°C, 0.5% methanol; 2–20°C, 1% methanol; 3–28°C, 0.5% methanol; 4–28°C, 1% methanol; 5–20°C, 0.5% methanol; 6–20°C, 1% methanol; 7–28°C, 0.5% methanol; 8–28°C, 1% methanol. (TIF) [file pone.0318459.s004.tif]

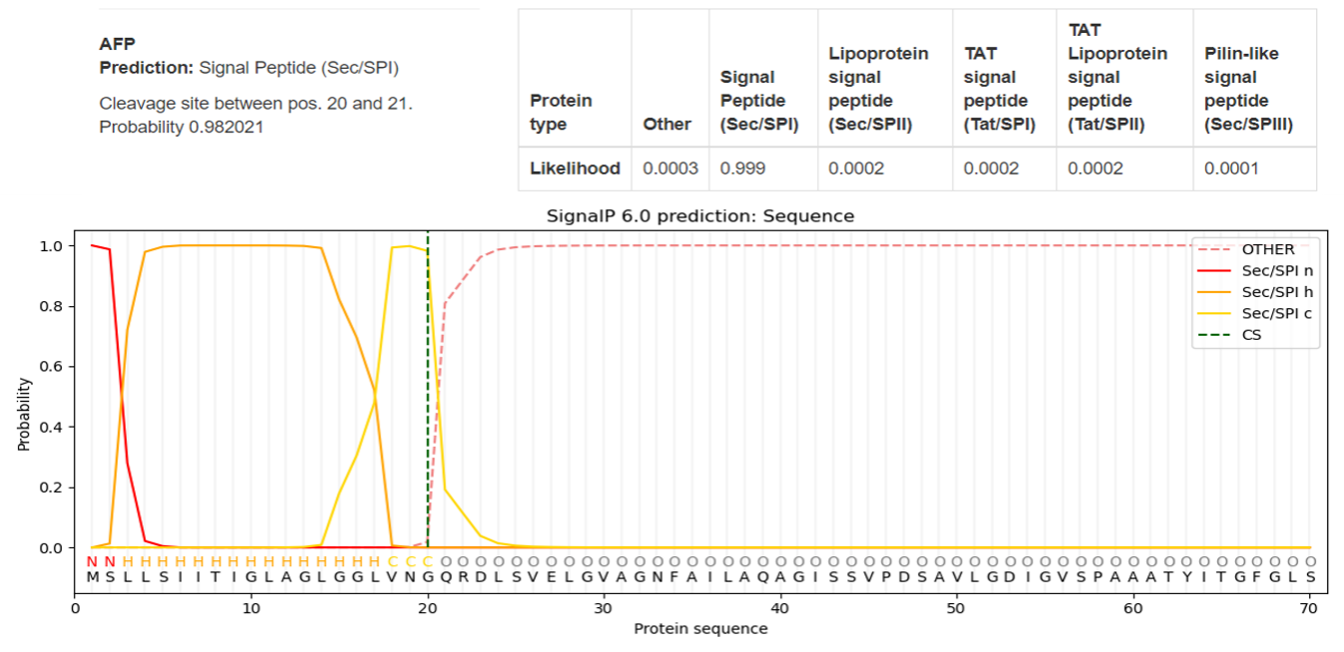

Supplement: S5 Fig — The SignalIP 6.0 bioinformatics tool confirmed the presence of a signal peptide enabling protein secretion in the first 20 amino acids of the expressed GaAFP protein with a probability of 0.999. Additionally, a cleavage site (CS) between the 20th and 21st amino acids was identified. (TIF) [file pone.0318459.s005.tif]

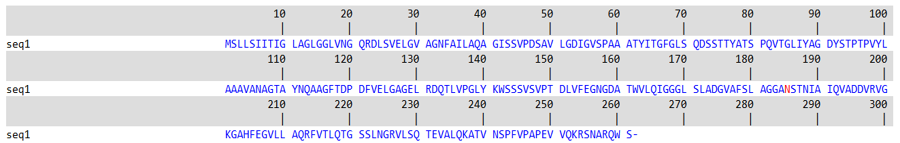

Supplement: S6 Fig — The likely site of glycosylation in GaAFP is asparagine at position 185. (TIF) [file pone.0318459.s006.tif]

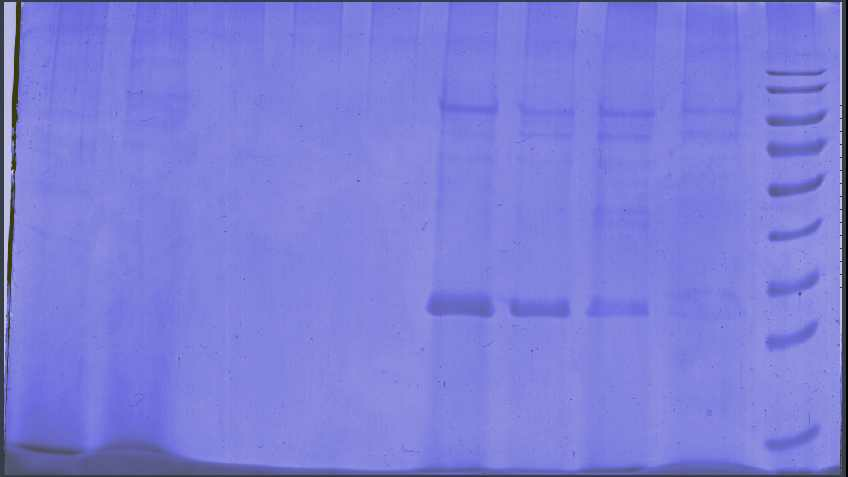

Supplement: S7 Fig — SDS-PAGE analysis, lanes: 1–supernatant after 72 h expression at 28°C with 1% of methanol induction, 2–post-culture liquid concentrated using tangential flow filtration, 3–fraction after ice affinity chromatography. (TIF) [file pone.0318459.s007.tif]
