# Supplementary material for: Antifreeze proteins produced by Antarctic yeast from the genus Glaciozyma as cryoprotectants in food storage
Source: PLoS One. 2025 Mar 6;20(3):e0318459. doi: 10.1371/journal.pone.0318459 (PMC11884722; doi:10.1371/journal.pone.0318459)
Supplement: S1 Table — (PDF) [file pone.0318459.s008.pdf]

**Sequence of synthetic *afp* gene from *Glaciozyma antarctica* PI12**
ATGTCCCTGTTGTCCATTATTACCATTGGTTTGGCTGGTTTAGGTGGTTTGGTTAATGGTC<br>AAAGAGATTTGTCTGTTGAATTGGGTGTTGCTGGTAACTTTGCTATTTTGGCTCAAGCTG<br>GTATTTCCTCTGTTCCAGATTCTGCTGTTTTGGGTGATATTGGTGTTTCTCCAGCTGCTGC<br>TACTTACATTACTGGTTTTGGTTTGTCCCAAGATTCTTCTACTACTTACGCTACTTCTCCAC<br>AAGTTACCGGTTTGATATATGCTGGTGATTACTCTACTCCAACTCCAGTTTATTTGGCTGC<br>TGCTGTTGCTAATGCTGGTACTGCTTATAATCAAGCTGCTGGTTTTACTGATCCAGACTTC<br>GTTGAATTAGGTGCTGGTGAATTGAGAGATCAAACTTTGGTTCCAGGTCTGTACAAATG<br>GTCATCTTCTGTTTCTGTTCCAACCGACTTGGTTTTTGAAGGTAATGGTGATGCTACCTG<br>GGTTTTACAAATTGGTGGTGGTTTGTCTTTGGCAGATGGTGTTGCTTTTTCATTGGCTGGT<br>GGTGCTAACTCTACCAACATTGCTATTCAAGTTGCCGATGATGTTAGAGTTGGTAAGGGT<br>GCTCATTTCGAAGGTGTTTTGTTGGCTCAAAGATTCGTTACCTTGCAAACTGGTTCTTCA<br>TTGAACGGTAGAGTTTTGTCTCAAACTGAAGTTGCCTTGCAAAAGGCTACTGTTAATTCT<br>CCATTTGTTCCAGCTCCAGAAGTC<br>GTCCAAAAAAGATCTAATGCTAGACAGTGGTCCTAA
**Amino acids sequence of GaAFP protein**
MSLLSIITIGLAGLGGLVNGQRDLSVELGVAGNFAILAQAGISSVPDSAVLGDIGVSPAAATYI<br>TGFGLSQDSSTTYATSPQVTGLIYAGDYSTPTPVYLAAAVANAGTAYNQAAGFTDPDFVELG<br>AGELRDQTLVPGLYKWSSSVSVPTDLVFEGNGDATWVLQIGGGLSLADGVAFSLAGGANST<br>NIAIQVADDVRVGKGAHFEGVLLAQRFVTLQTGSSLNGRVLSQTEVALQKATVNSPFVPAPE<br>VVQKRSNARQWS-
The predicted molecular mass based on the sequence is 26.57 kDa, and the isoelectric point is 4.4.
