# Supplementary material for: Antifreeze proteins produced by Antarctic yeast from the genus Glaciozyma as cryoprotectants in food storage
Source: PLoS One. 2025 Mar 6;20(3):e0318459. doi: 10.1371/journal.pone.0318459 (PMC11884722; doi:10.1371/journal.pone.0318459)
Supplement: S2 Table — (PDF) [file pone.0318459.s009.pdf]

Name of primer	Sequence
FWDEcoRIpPICZ	GCGCGAATTCATGTCCCTGTTGTCCATTATT
REVKpnIpPICZ	CGATGGTACCTTAGGACCACTGTCTAGCATT
5'AOX1	GACTGGTTCCAATTGACAAGC
3'AOX1	GCAAATGGCATTCTGACATCC
