# Supplementary material for: Antifreeze proteins produced by Antarctic yeast from the genus Glaciozyma as cryoprotectants in food storage
Source: PLoS One. 2025 Mar 6;20(3):e0318459. doi: 10.1371/journal.pone.0318459 (PMC11884722; doi:10.1371/journal.pone.0318459)
Supplement: S3 Table — (PDF) [file pone.0318459.s010.pdf]

Sample variant with time of storage		Minimal size of ice crystals [µm]	Maximal size of ice crystals [µm]	Average Diameter in the Class with the Highest Frequency ±SD [µm]
**Negative - Sucrose**	**0 min**	2.45	13.10	8.58±1.34
	**10 min**	6.33	17.22	10.70±1.71
	**20 min**	5.74	21.89	13.65±2.74
	**30 min**	7.61	24.43	14.55±3.09
	**40 min**	10.34	35.76	19.06±3.57
	**50 min**	13.21	30.57	20.67±3.09
	**60 min**	17.15	55.10	32.58±3.75
**GmAFP**	**0 min**	3.85	12.68	8.11±1.54
	**10 min**	5.16	13.32	8.88±1.20
	**20 min**	5.10	12.08	9.01±1.19
	**30 min**	4.56	13.63	8.90±1.22
	**40 min**	5.5	12.35	9.14±0.99
	**50 min**	6.98	13.51	9.76±1.33
	**60 min**	6.71	18.14	11.69±1.85
**GaAFP**	**0 min**	2.36	9.73	5.40±0.94
	**10 min**	4.72	8.20	6.35±0.67
	**20 min**	4.35	11.64	6.95±1.29
	**30 min**	1.96	12.95	8.59±1.55
	**40 min**	3.71	12.77	9.20±1.48
	**50 min**	6.53	17.47	12.25±1.93
	**60 min**	7.42	17.59	12.53±1.73
