# Supplementary material for: Antifreeze proteins produced by Antarctic yeast from the genus Glaciozyma as cryoprotectants in food storage
Source: PLoS One. 2025 Mar 6;20(3):e0318459. doi: 10.1371/journal.pone.0318459 (PMC11884722; doi:10.1371/journal.pone.0318459)
Supplement: S4 Table — Among the selected species with the highest percentage of identity to the tested sequence (ITS region and D1/D2 domain) were: Glaciozyma martinii, Glaciozyma antarctica, and Glaciozyma watsonii. Glaciozyma martinii exhibited the highest percentage of identity (over 99%), with sequence coverage for the selected phylogenetic marker close to 100% and minimal gaps. Glaciozyma antarctica displayed a lower percentage of identity at approximately 94% and a higher content of gaps (2%–3%) with coverage close to 100%. Glaciozyma watsonii showed a similar situation to Glaciozyma antarctica. Phenoliferia himalayensis exhibited a much lower identity at 89.39% with nearly 100% coverage. The sequence identity of Rhodotorula svalbardensis was at 82.89% with a sequence coverage value of 85%. Zymoxenogloea eriophori demonstrated the lowest identity with 100% sequence coverage. From the listed sequences, those suitable for phylogenetic analysis using the MEGA11 software were selected. (PDF) [file pone.0318459.s011.pdf]

**Microorganism**	**Accession number**	**Coverage of the query sequence**	**Sequence length (bp)**	**Percentage of identity**	**Gaps**
Glaciozyma martinii 54A-1	LC202042	100%	1183	99.75% (1180/1183)	0% (3/1183)
Glaciozyma martinii 9A-10-1	LC203686	99%	1178	99.66% (1178/1182)	0% (4/1182)
Glaciozym amartinii 9A-10-2	LC203687	99%	1178	99.58% (1175/1180)	0% (5/1180)
Glaciozyma martinii 9A-10-3	LC203688	99%	1175	99.74% (1171/1174)	0% (3/1174)
Glaciozyma martinii 9A-10-4	LC203689	100%	1184	99.66% (1179/1183)	0% (4/1183)
Glaciozyma martinii 9A-4-1	LC203681	99%	1179	99.58% (1173/1178)	0% (5/1178)
Glaciozyma martinii 9A-4-2	LC203682	99%	1177	99.75% (1174/1177)	0% (3/1177)
Glaciozyma martinii 9A-4-3	LC203683	99%	1178	99.74% (1171/1174)	0% (3/1174)
Glaciozyma martinii 9A-4-4	LC203684	99%	1177	99.58% (1177/1182)	0% (5/1182)
Glaciozyma martinii 9A-4-5	LC203685	99%	1176	99.75% (1174/1177)	0% (3/1177)
Glaciozyma watsonii A2P-1	MH481689	99%	1204	94.18% (1132/1202)	2% (28/1202)
Glaciozyma watsonii A4-2	MH481674	100%	1208	93.96% (1135/1208)	2% (30/1208)
Glaciozyma watsonii A4P-11	MH481682	99%	1204	94.16% (1129/1199)	2% (28/1199)
Glaciozyma watsonii G1-4-4	LC514930	99%	1193	93.99% (1126/1198)	2% (30/1198)
Glaciozyma watsonii P4(2)	MH481697	99%	1200	93.99% (1127/1199)	2% (30/1199)
Phenoliferia himalayensis 4B	AM410636	99%	1193	89.39% (1061/1187)	3% (38/1187)
Rhodotorula sp. *svalbardensis* J-174	KY782282	85%	1163	92.89% (941/1013)	0% (9/1013)
Glaciozyma antarctica PI12 UPM	FJ554838	100%	1263	94.22% (1145/1214)	2% (33/1214)
Glaciozyma antarctica 54A-11	LC202043	99%	1217	94.22% (1142/1212)	3% (37/1212)
Glaciozyma antarctica CBS 5942	AF444529	100%	1279	93.11% (1149/1234)	2% (53/1234)
Zymoxenogloea eriophori CBS 8387	AF444602	100%	1223	88.88% (1063/1196)	2% (33/1196)
